# Psychotropic Drug Scheduling and Benzodiazepine Receptor Agonist Prescribing in Japan: An Interrupted Time Series Analysis

**DOI:** 10.1007/s43441-026-01029-4

**Published:** 2026-08-08

**Authors:** Kota Tatsuoka, Shota Hamada, Sayuri Shimizu, Makoto Tomita

**Affiliations:** 1 School of Data Science, Graduate School of Data Science, Yokohama City University, Yokohama, Japan; 2 Department of Pharmacoepidemiology, School of Pharmacy, Tokyo University of Pharmacy and Life Sciences, 1432-1 Horinouchi, Hachioji, Tokyo, 192-0392 Japan; 3 Research Department, Association for Health Economics Research and Social Insurance and Welfare, Institute for Health Economics and Policy, Tokyo, Japan

**Keywords:** Benzodiazepines, Legislation, Psychotropic drug scheduling, Z-drugs

## Abstract

**Background:**

This study aimed to evaluate the effects of psychotropic drug scheduling, including limiting the days’ supply per prescription, on the prescriptions of etizolam and zopiclone, benzodiazepine receptor agonists (BZRAs), in Japan.

**Methods:**

This study was based on the randomly sampled data from the national administrative health insurance claims data in Japan from 2012 to 2020. We analyzed age-specific trends in prescriptions of the two BZRAs, which were designated as psychotropic drugs in 2016. The impacts of psychotropic drug scheduling on the prescription trends were evaluated using interrupted time series analysis.

**Results:**

After the scheduling, etizolam prescriptions showed an immediate decrease and a continuing decrease at a greater rate than before the intervention in patients aged 80 years or older (Level [per 10,000 people], −22.23, 95% CI −39.57, −4.88; Slope [per 10,000 people / 3 months], −8.07, 95% CI −10.14, −6.00). Among patients aged 80 years or older, the prescription of zopiclone decreased immediately after the scheduling, with no trend change (Level [per 10,000 people], −7.20, 95% CI −11.99, −2.42; Slope [per 10,000 people / 3 months], −0.04, 95% CI −0.61, 0.53). Changes in the other age groups were generally smaller than those observed in the ≥ 80 years group.

**Conclusions:**

Following the psychotropic scheduling, significant decreases in the prescription rates of etizolam and zopiclone were observed among patients aged 80 years or older. The effects of psychotropic drug scheduling of BZRAs on reducing prescriptions were modest and may be insufficient to substantially influence prescribing behavior, suggesting the need for additional or complementary approaches, such as clinician education and stricter medication review.

## Introduction

Benzodiazepine receptor agonists (BZRAs), including benzodiazepines and non-benzodiazepine hypnotics (Z-drugs), have been widely used as pharmacotherapy for anxiety and insomnia. However, these drugs carry risks of adverse events, such as falls, fractures, and dependence, especially by the long-term use [1]. Nevertheless, BZRA consumption remains high worldwide including Japan [2].

In several countries, governments introduced legal or regulatory measures to address inappropriate use of BZRAs by facilitating the changes of clinical practice. Governmental initiatives have the potential for substantial population-level impact because of the application to a broad range of patient populations and care settings. In Ontario, Canada, the introduction of a legal framework, including requiring prescribers to record specified information and dispensers to verify them in 2011, resulting in reduced prescription rates of benzodiazepines but the effect was small in older adults [3]. In Australia, changes including reducing the pack size of alprazolam, eliminating refills, and delisting public subsidy of high-dose tablet were implemented in 2017, resulting in a decrease in total dispensing [4]. Meanwhile, in Ireland, prescription requirements of BZRAs were tightened in 2017, there was no significant impact although the prescription rate for those aged 65 or older continued to decline [5]. Therefore, various attempts to reduce the inappropriate use of BZRAs implemented in some countries had achieved limited success.

In Japan, legal regulations on psychotropic drugs were expanded in 1990 following the ratification of the 1971 Convention on Psychotropic Substances. Most BZRAs are designated as controlled substances, which are subject to stricter distribution controls and the limited days’ supply per prescription. Etizolam and zopiclone were finally designated as controlled psychotropic substances in 2016, with 30-day supply limit per prescription, about 30 years after their launch in Japan. Until now, the impact of this scheduling on prescribing has not been well studied.

Apart from the scheduling, in Japan, medical fees are sometimes revised in order to change clinical practice. Revisions to the medical fees were implemented several times during the 2010s to reduce the prescription of and long-term use of BZRAs; however, these measures had no significant effect on long-term prescriptions of BZRAs [6, 7].

Therefore, effective measures to facilitate appropriate use of BZRAs remains limited. This study aimed to evaluate the effects of psychotropic drug scheduling, including limiting the days’ supply per prescription, on the prescriptions of the scheduled drugs, based on the examples of etizolam and zopiclone in Japan.

## Methods

### Data Source

The National Database of Health Insurance Claims and Specific Health Checkups of Japan (NDB) contains almost all the administrative health insurance claims nationwide. The NDB Sampling Dataset, which consists of anonymized, stratified random samples of the NDB in January, April, July and October each year, are prepared for research. Furthermore, recently, aggregated data using the dataset has been publicly available by the Initiative for Clinical Epidemiological Research [8], and some studies using this data have contributed to literature [9]. Among the latest dataset (version 0.2), this study used the data on the number of outpatients prescribed BZRAs and other hypnotics from January 2012 to January 2020, obtained from a 1% age-stratified random sample of the NDB prescriptions.

### Drugs of Interest

The drugs of interest included etizolam (an anxiolytic benzodiazepine) and zopiclone (a Z-drug). These drugs were designated as psychotropic substances on October 14, 2016. The rule limiting prescriptions to a 30-day supply under health insurance coverage was issued on October 13 and took effect on November 1, 2016. In addition, we evaluated as reference the prescribing trends on some frequently prescribed anxiolytic and hypnotic BZRAs which had already been designated as psychotropic drugs at the start of the study period, and newer types of hypnotics, which were introduced during the study period and were not designated as psychotropic drugs.

### Statistical Analysis

We analyzed the number of monthly outpatient prescription rates across Japan for each drug by age groups (15–39, 40–64, 65–79, and ≥ 80 years) to assess whether the effect of the regulation on prescribing patterns varied across age groups. The age categories were defined to broadly reflect life stages and potential clinical heterogeneity. The analyses were conducted across all the age groups because the prescribing restriction applied to the entire population, and no primary age group was prespecified. Prescription rates (people receiving at least one per month per 10,000 population) were calculated for each age group based on the number of patients from the dataset, the sampling rate of data, and the age-specific population obtained from the e-Stat website (https://www.e-stat.go.jp/). To examine the impact of psychotropic drug scheduling in October 2016 (30-day supply limit per prescription, applicable to prescriptions issued on or after November 1, 2016), we applied interrupted time series analysis from January 2012 to January 2020 (33 time points). Interrupted time series analysis is a quasi-experimental approach used to evaluate interventions in populations [10]. Regression analysis estimates the change in slope (trend change) and change in level immediately following the intervention [10]. The intervention point was set as January 2017, taking into account the effective date of prescribing restrictions.

The estimation formula is: ${Y}_{t}={\beta}_{0}+{\beta}_{1}T+{\beta}_{2}{X}_{t}+{\beta}_{3}\left(T-{T}_{i}\right){X}_{t}$ where Y_t_ is the prescription rate at a time point (t), ${\beta}_{0}$ is the baseline level at T = 0, ${\beta}_{1}$ represents baseline rate of change, ${\beta}_{2}$ is the level change, ${\beta}_{3}$ indicates the slope change which affects after the intervention (${X}_{t}=1,t\geq t_{i}$).

Seasonality was adjusted by removing the seasonal component extracted by the seasonal-trend decomposition using loess [11]. The seasonally adjusted data were analyzed with a linear model that included both slope and level changes, and point estimates with 95% confidence intervals (CI) were calculated. Residual autocorrelation was evaluated using autocorrelation function. Analyses were performed using R-4.4.1.

## Results

**Fig. 1:**
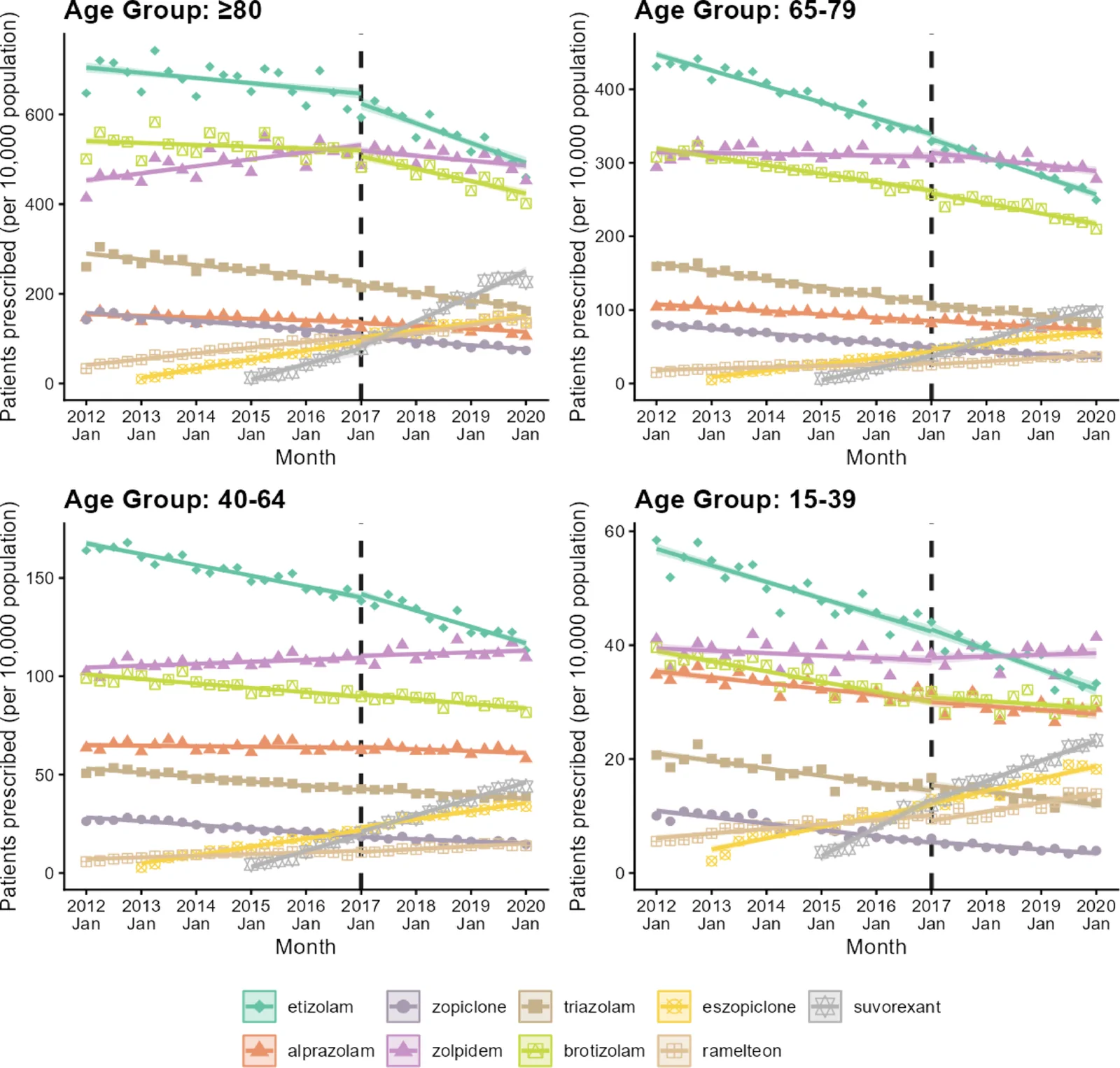
Trends in prescription rates by age group. Number of people who received at least one prescription per month per 10,000 people. The vertical dashed line represents the intervention time point in analysis (January 2017). The y-axis scales differ across age groups.

Prescription trends for BZRAs and other hypnotics by age groups are shown in the Fig. 1. The autocorrelation function of the model residuals fell within the 95% CI. Overall, prescription rates of most conventional BZRAs, except zolpidem, had been decreased before the scheduling of etizolam and zopiclone in October 2016. The prescription rate of zopiclone also decreased, whereas zolpidem prescriptions increased among patients aged 80 years or older, and triazolam and brotizolam showed decreasing or flat trends. The prescription rates of newer hypnotics, including eszopiclone, ramelteon, and suvorexant, continued to increase across all age groups.

After the scheduling, etizolam prescriptions among patients aged ≥ 80 years represents an immediate decrease and continued to decline at a faster rate compared to the period before the intervention (Level [per 10,000 people], −22.23, 95% CI −39.57, −4.88; Slope [per 10,000 people / 3 months], −8.07, 95% CI −10.14, −6.00) (Table 1). Among those aged < 80 years, a significant decrease in slope was observed for patients aged 40–64 years (−0.71, 95% CI −1.01, −0.41), with no significant changes in other age groups. A significant decrease in slope was also observed for alprazolam, a reference anxiolytic BZRA (−0.79, 95% CI −1.33, −0.26).

**Table 1:** Changes in prescribing rates after psychotropic scheduling for etizolam and zopiclone

Drug	Age group	Slope change	(95% CI)	P value	Level change	(95% CI)	*P* value
*1. Anxiolytics*							
(1) Benzodiazepines							
Etizolam	≥ 80	−8.07	(−10.14, −6.00)	< 0.01	−22.23	(−39.57, −4.88)	0.02
	65–79	−0.85	(−1.81, 0.11)	0.09	−6.38	(−14.42, 1.66)	0.13
	40–64	−0.71	(−1.01, −0.41)	< 0.01	1.66	(−0.83, 4.16)	0.20
	15–39	−0.15	(−0.34, 0.03)	0.11	0.35	(−1.18, 1.88)	0.66
Alprazolam	≥ 80	−0.79	(−1.33, −0.26)	< 0.01	−2.50	(−7.02, 2.01)	0.29
	65–79	0.07	(−0.25, 0.38)	0.68	0.80	(−1.83, 3.43)	0.55
	40–64	−0.17	(−0.41, 0.06)	0.15	0.30	(−1.63, 2.23)	0.76
	15–39	0.08	(−0.06, 0.21)	0.28	−0.26	(−1.40, 0.88)	0.65
*2. Hypnotics*							
(1) Z-drugs							
Zopiclone	≥ 80	−0.04	(−0.61, 0.53)	0.90	−7.20	(−11.99, −2.42)	< 0.01
	65–79	0.66	(0.42, 0.90)	< 0.01	−1.62	(−3.62, 0.39)	0.13
	40–64	0.21	(0.06, 0.35)	< 0.01	−0.28	(−1.47, 0.91)	0.65
	15–39	0.12	(0.03, 0.21)	0.01	0.09	(−0.65, 0.84)	0.81
Zolpidem	≥ 80	−6.72	(−8.14, −5.30)	< 0.01	−12.69	(−24.59, −0.79)	0.05
	65–79	−1.76	(−2.72, −0.80)	< 0.01	5.06	(−2.98, 13.10)	0.23
	40–64	−0.02	(−0.27, 0.23)	0.88	1.05	(−1.04, 3.14)	0.33
	15–39	0.18	(0.02, 0.34)	0.03	0.42	(−0.89, 1.74)	0.53
(2) Benzodiazepines							
Triazolam	≥ 80	−1.05	(−1.99, −0.11)	0.04	−6.82	(−14.72, 1.07)	0.10
	65–79	0.89	(0.42, 1.36)	< 0.01	−1.26	(−5.21, 2.68)	0.54
	40–64	0.14	(−0.01, 0.29)	0.07	0.68	(−0.55, 1.91)	0.29
	15–39	0.04	(−0.09, 0.17)	0.52	0.99	(− 0.08, 2.06)	0.08
Brotizolam	≥ 80	−5.87	(−7.43, −4.31)	< 0.01	−13.25	(−26.30, −0.20)	0.06
	65–79	−0.59	(−1.38, 0.20)	0.15	−3.45	(−10.05, 3.16)	0.32
	40–64	−0.02	(−0.28, 0.24)	0.87	1.19	(−0.99, 3.36)	0.29
	15–39	0.29	(0.16, 0.42)	< 0.01	0.81	(−0.27, 1.88)	0.15
(3) Newer types							
Eszopiclone	≥ 80	−1.06	(−1.79, −0.33)	< 0.01	8.34	(2.44, 14.24)	0.01
	65–79	−0.09	(−0.34, 0.15)	0.45	2.83	(0.87, 4.79)	< 0.01
	40–64	−0.02	(−0.17, 0.13)	0.80	1.51	(0.29, 2.74)	0.02
	15–39	0.05	(−0.07, 0.18)	0.39	0.23	(−0.76, 1.22)	0.65
Ramelteon	≥ 80	1.10	(0.33, 1.88)	< 0.01	−10.69	(−17.19, −4.18)	< 0.01
	65–79	0.58	(0.30, 0.86)	< 0.01	−1.87	(−4.18, 0.44)	0.12
	40–64	0.14	(0.01, 0.26)	0.04	−0.63	(−1.67, 0.42)	0.25
	15–39	0.20	(0.09, 0.31)	< 0.01	−0.97	(−1.90, −0.03)	0.05
Suvorexant	≥ 80	5.31	(2.19, 8.42)	< 0.01	3.94	(−13.17, 21.04)	0.66
	65–79	1.42	(0.31, 2.53)	0.02	−0.82	(−6.89, 5.25)	0.79
	40–64	0.03	(−0.34, 0.41)	0.86	1.60	(−0.44, 3.63)	0.14
	15–39	−0.36	(−0.61, −0.10)	0.01	−0.33	(−1.74, 1.08)	0.65

CI, confidence interval

Among patients aged ≥ 80 years, zopiclone prescriptions decreased immediately after the scheduling, without a significant slope change (Level [per 10,000 people], −7.20, 95% CI −11.99, −2.42; Slope [per 10,000 people / 3 months], −0.04, 95% CI −0.61, 0.53). In contrast, in those aged < 80 years, slopes increased after the scheduling. For other hypnotic BZRAs, except for eszopiclone, significant decreases in level or slope were observed in patients aged ≥ 80 years.

## Discussion

In this study using the nationally representative sample, after the psychotropic drug scheduling, in patients aged 80 years or older, etizolam prescriptions showed a continued additional decrease and zopiclone prescriptions showed an immediate decrease. The observed reductions in this age group may suggest the scheduling led to some changes in treatment strategies, such as no use, discontinuation, or switching. In contrast, there was no significant change or a slowing downward trend in patients aged < 80 years. These results mean that psychotropic scheduling had a modest impact on the prescribing of BZRAs for very old people. The findings of this study are consistent with previous reports that legislative interventions have achieved only limited improvements in BZRA prescribing [3–5]. Even for clinical interventions, changing clinicians’ prescribing practices is challenging [12].

Although the underlying mechanisms could not be directly examined in this study, several factors may explain the age-specific effects. Very old people are more likely to have frailty and multimorbidity and receive polypharmacy, which may make their prescribing patterns more responsive to interventions. In addition, more frequent healthcare encounters in this population may provide greater opportunities for medication review. Furthermore, these changes in prescriptions could be partly attributed to the publication of clinical practice guidelines and national guidance on pharmacotherapy for older adults to avoid the inappropriate prescribing in 2015 and 2016, respectively [13, 14]. These likely enhanced awareness of appropriate use or deprescribing of BZRAs and influenced prescribing patterns.

A strength of the present study is that prescribing trends were assessed comprehensively, including alternative drugs. In fact, during the study period, prescription rates of most BZRAs decreased, whereas prescriptions for eszopiclone, ramelteon and suvorexant increased, consistent with a possible shift from conventional BZRAs to newer hypnotics. The increase in newer hypnotics, particularly suvorexant, was comparable in magnitude to the decrease in conventional BZRAs. The shifts from conventional BZRAs to newer hypnotics were also reported in acute care hospitals [15].

There are some limitations in this study. First, we could not assess new initiations, discontinuations, persistence, switches to other medications, concomitant use of multiple types of anxiolytics and hypnotics, dose, days supplied, and clinical outcomes in this study because of the use of aggregated prescription data every 3 months. In addition, we could not account for individual-level factors such as sex, comorbidities, or prescriber specialty, which may influence prescribing decisions. Studies using patient-level data are needed to clarify prescription trajectories and related factors. Second, the dataset cannot examine the number of visits, which may increase after days’ supply restriction, and prescription dosage or total drug consumption. This made it difficult to completely understand the effects on clinical practice or healthcare-seeking behavior. It should be also acknowledged that the decreases in the number of prescriptions do not always decrease the total amount of drugs prescribed, as reported in Australia [6]. Third, the study period was determined by the time span available in the dataset. A longer observation period may provide insights into long-term effects and temporal fluctuations, although extending the period may increase susceptibility to confounding from other concurrent changes in clinical practice or policy. Fourth, because analyses were performed across multiple age groups and included both the drugs targeted by the prescribing restriction and reference drugs for comparison, without adjustment for multiplicity, some statistically significant findings may have occurred by chance and should be interpreted with caution. Finally, the effects of similar interventions may not be generalizable to other settings because of differences in the prevalence of target drugs and alternatives or diseases and medical environments including laws.

## Conclusions

Following the psychotropic scheduling, significant decreases in etizolam and zopiclone prescription rates were observed among patients aged ≥ 80 years. These changes may be relevant to medication-safety efforts, but whether scheduling reduced adverse events requires patient-level outcome studies. However, other age groups showed either no notable change or a more gradual decrease. Overall, the effects of psychotropic scheduling of BZRAs on reducing prescriptions were modest and may be insufficient to substantially influence prescribing behavior, suggesting the need for additional or complementary approaches, such as clinician education and stricter medication review.

## Data Availability

The data used for this study are publicly available on the website of the Initiative for Clinical Epidemiological Research at [https://icer.tokyo/materials/ndb\_feasibility\_check/](https:/icer.tokyo/materials/ndb_feasibility_check).
